# Insights into post‐polymerisation modification of bio‐based unsaturated itaconate and fumarate polyesters via aza‐michael addition: Understanding the effects of C=C isomerisation

**DOI:** 10.1002/pola.29079

**Published:** 2018-08-09

**Authors:** T. J. Farmer, D. J. Macquarrie, J. W. Comerford, A. Pellis, J. H. Clark

**Affiliations:** ^1^ Department of Chemistry, Green Chemistry Centre of Excellence University of York Heslington York YO10 5DD United Kingdom

**Keywords:** biopolymers and renewable polymers; functionalization of polymers; monomers; oligomers and telechelics; polycondensation; resins

## Abstract

Development of renewable bio‐based unsaturated polyesters is undergoing a renaissance, typified by the use of itaconate and fumarate monomers. The electron‐deficient C=C bond found on the corresponding polyesters allows convenient post‐polymerisation modification to give a wide range of polymer properties; this is notably effective for the addition of nucleophilic pendants. However, preservation of unsaturated functionality is blighted by two undesirable side‐reactions, branching/crosslinking and C=C isomerisation. Herein, a tentative kinetic study of diethylamine addition to model itaconate and fumarate diesters highlights the significance of undesirable C=C isomerisation. In particular, it shows that reversible isomerisation from itaconate to mesaconate (a poor Michael acceptor) is in direct competition with aza‐Michael addition, where the amine Michael donor acts as an isomerisation catalyst. We postulate that undesired formation of mesaconate is responsible for the long reaction times previously reported for itaconate polyester post‐polymerisation modification. This study illustrates the pressing need to overcome this issue of C=C isomerisation to enhance post‐polymerisation modification of bio‐based unsaturated polyesters. © 2018 Wiley Periodicals, Inc. J. Polym. Sci., Part A: Polym. Chem. **2018**, *56*, 1935–1945

## INTRODUCTION

**Scheme 1:**
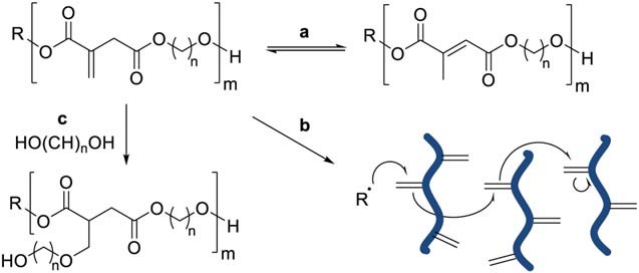
Typical undesired side‐reactions of bio‐based unsaturated polyesters (UPEs) (A) isomerisation of unsaturated unit, (B) radical induced crosslinking via C=C, (C) Ordelt saturation (oxo‐Michael addition of —OH end‐group onto C=C) inducing crosslinking. [Color figure can be viewed at http://wileyonlinelibrary.com]

Over the last decade there has been a growing interest in the utilisation of bio‐derived platform molecules for the synthesis of higher value products, triggered for the most part by the US DOE report on Top Value Added Chemicals from Biomass.^1^ An area of the particular interest has been in the field of polymer synthesis using these sustainably sourced building blocks as monomers or monomer precursors. Plastics such as poly(lactic acid) (PLA), poly(butylene succinate) (PBS), and poly(ethylene furanoate) (PEF) demonstrate how polymers with favourable properties can be partly or wholly derived from platform molecules.^2^ More recently there has been increasing focus toward functionalisable polymers and in particular, the polymerisation of common platform molecules, itaconic acid, and fumaric acid with a range of diols such as 1,2‐ethanediol, 1,2‐propanediol, 1,3‐propanediol, 1,4‐butanediol, and glycerol and to produce novel, 100% bio‐derived unsaturated polyester resins (UPEs).^3^ Synthesis of these polymers typically employs well‐established melt polymerisation methods along with well‐known metal‐centred catalysts (Ti, Al, Sn, Zn).^4^ However, due to the unsaturated nature of the dicarboxylates, they often undergo undesired side reactions such as isomerisation, radical crosslinking, and Ordelt saturation (an oxo‐Michael addition, where an R—OH end‐group attack the conjugated C=C through a β‐addition, [Scheme 1(C)].^4^ ^(a,b)^

**Scheme 2:**
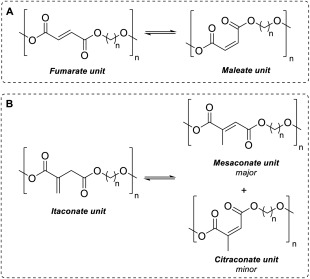
Isomerisation between (A) fumarate and maleate UPEs and (B) itaconate and mesaconate/citraconate UPEs.

Radical crosslinking for the most part can be quenched via the use of scavengers such as quinol^4^ ^(f)^ and 4‐methoxyphenol,^4^ ^(b)^ while significantly less has been done to limit Ordelt saturation. This is largely due to the fact that typical acid catalysts used to promote the polytransesterification, also increases the ability of the conjugated C=C to act as a Michael acceptor to a hydroxyl end‐group. A very recent study by Robert and coworkers found that Lewis acidic Zn(OAc)_2_ gave the lowest gelation, while Brønsted acid catalysts such as methanesulfonic acid made gelation considerably worse.^4^ ^(b)^ Crosslinking of itaconate UPEs tends to give soft, rubber like polymers suitable only for applications that do not require inherent strength such as coatings,^5^ shape memory polymers,^6^ elastomers,^7^ composites,^4^ ^(c)^ and medical applications such as bio‐erodible vaccine loaded hydrogels.^8^ Branching and eventual crosslinking also limits the solubility of the resulting UPEs and this may affect downstream processing including formulation or post‐polymerisation modification. Undesirable isomerisation of the C=C is also widely reported for UPEs of itaconate, fumarate, and maleate monomers, where the latter two can interchange between one another, [Scheme 2(A)]. In the case of itaconate containing polyesters, regioisomerisation results in the formation of mesaconate (major) and citraconate (minor) units, [Scheme 2(B)]. Formation of these regio‐isomer units lead to greater complexity in the analysis of the polyesters, whilst also effecting reproducibility of the polymers final thermal and mechanical properties.

Several studies report regioisomerisation of itaconate during polyester synthesis, ranging from <10% (relative to itaconate) as described by Spasojevic (7%),^9^ Farmer (8%),^4^ ^(a)^ and Teramoto (9%),^10^ increasing up to nearly 60% in the Takasu's protocol using itaconic anhydride and 1,2‐epoxybutane with a magnesium ethoxide initiator.^11^ Regioisomerisation has additionally been observed to occur during addition of dicarbonyl pendants to free unsaturated sites; with an increasing ratio of mesaconate seen for high (>75%) but not complete addition.^12^ Indeed, several recent studies have demonstrated post‐polymerisation modification (PPM) of bio‐based UPEs, allowing these polyester backbones to be altered via facile Michael additions.^3^ Examples now report the addition of thiols, amines, and chelating 1,3‐dicarbonyls to bio‐based UPEs, resulting in polyesters with tailored properties and a wider range of potential applications.^4^ ^(f)^,^12^, ^13^ However, PPM of highly crosslinked and isomerised UPEs is still problematic as the polymer needs to remain soluble during the addition whilst the isomerised units likely have differing rates of accepting the adduct. Intriguingly, in our previous investigations we observed that despite the itaconate UPEs containing isomerised mesaconate moieties (∼7% relative to itaconate) prior to PPM, the eventual 100% pendanted polyesters only had a single constitutional β‐substituted repeat unit.^12^ This implies that only the itaconate form is able to act as a Michael acceptor and the mesaconate must first isomerises back to itaconate prior to pendant addition. Although this observation has not been specifically described elsewhere it is clear from the other published studies that very long reaction times and large excesses of donor are commonplace, likely a result of the undesired isomerisation reducing significant the rate of reaction. Lv et al. reported the need for 14–20 h reaction time for addition of thiols and amines, though the Michael donors were used in a 15‐times molar excess.^13^ Ramakrishnan reported the need for 3 days for the addition of both thiols and amines to their poly(dodecyl itaconate) UPEs,^4^ ^(f)^ while Meier suggested their thio‐Michael additions ran overnight, using a 5‐times molar excess of donor and included the 10%mol hexylamine as a catalyst.^4^ ^(e)^

Clearly, there remains a lack of understanding as to the degree of isomerisation occurring in the synthesis and PPM of itaconate and fumarate based polymers, as well as the change in equilibrium between the different isomers. This is despite the fact that the isomerisation must clearly effect the rate at which the addition can take place during PPM. Initial efforts have been made to overcome crosslinking and isomerisation via the use of enzymes under milder reaction conditions, although it was found that 1,4‐butanediol was an unsuitable monomer, and instead a more rigid cyclic diol was required for successful oligomerisation.^14^ As such there is evidently a need to overcome these issues in the chemocatalytic system. Herein, we describe a series of experiments to gain a detailed insight into the reaction pathway and kinetics of the isomerisation and aza‐Michael addition steps. In doing so we aim to better understand how undesired side reactions can affect PPM of bio‐based itaconate UPEs, and highlight the pressing need to develop strategies to suppress its occurrence.

## RESULTS AND DISCUSSION

**Scheme 3:**
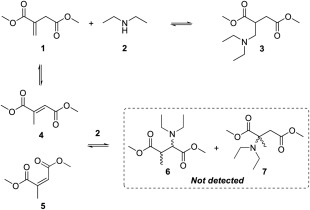
Aza‐Michael addition of DEA (**2**) onto DMI (**1**) and isomerisation of DMI (**1**) to dimethyl mesoconate (**4**) and dimethyl citraconate (**5**), with corresponding aza‐Michael addition products (**6** and **7**).

For simplicity and convenience investigation was initially focused on a model reaction of an aza‐Michael addition of diethylamine (DEA, **2**) to dimethyl itaconate (**1**), allowing rapid GC‐FID analysis and avoiding complications associated with using polymers such as changing viscosity and mass transfer and broad peaks in ^1^H NMR spectra. DEA (**2**) was selected as a readily available secondary amine, with a low boiling point (56 °C) allowing easy removal and analysis, whilst still having the potential to be sourced from biomass (via ethanol and ammonia). The resulting aza‐Michael product (**3**), along with the regioisomers dimethyl mesaconate (**4**), and dimethyl citraconate (**5)** and their subsequent aza‐Michael adducts (**6** and **7**) were monitored and, where observed, characterised by NMR spectroscopy and GC–MS (Scheme 3).

**Figure 1:**
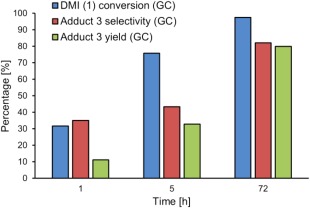
Initial time‐course determination for the % conversion of DMI, % selectivity and % yield to adduct **3** for the aza‐Michael addition between DMI and DEA. No catalyst, 21 °C, aza‐Michael addition of DEA to DMI. 2.5 mmol DMI, 8.2 mmol DEA. [Color figure can be viewed at http://wileyonlinelibrary.com]

Initially the aza‐Michael addition was performed without catalyst at 21 °C with a 3:1 ratio of DEA:DMI under solvent free conditions, as would be typical for additions onto UPEs or unsaturated monoesters (Fig. 1).^4^ ^(e,f),^ 13, 15 Reaction times of up to 72 h were required for yields above 80% of adduct **3**. However, throughout the reaction period it was clear that a portion of DMI (**1**) was isomerising to **4** (mesaconate), as seen with a similar previous study for the addition of acetylacetone to itaconate.^12^ Increasing the DEA:DMI ratio to 5:1 whilst extending the length of reaction to 5 days resulted in complete conversion of DMI and an 94% isolated yield of adduct **3**. ^1^H and ^13^C NMR spectroscopy of this crude product (Supporting Information Fig. S1) interestingly confirmed that the only aza‐Michael adduct detected was **3**, indicating complete regio‐selectivity. Aza‐Michael adducts **6** and **7** potentially formed from **4** were not observed, and only a small amount of residual mesaconate **4** was detected in the ^1^H NMR spectra (Supporting Information Fig. S1, top spectra).

Importantly, over long reaction times it appeared that the reaction resulted in complete regio‐selectivity to product **3** (i.e., addition to itaconate) even despite the possibility of Michael addition to regioisomer dimethyl mesaconate **4** to give products **6** and **7**.

### Effects of Altering DEA:DMI Ratio

**Figure 2:**
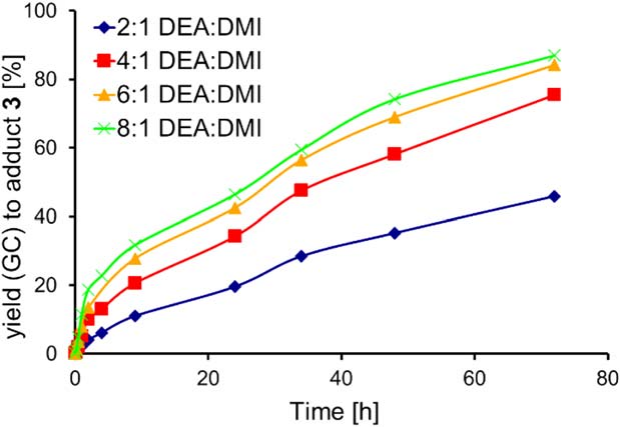
Effects of altering DEA:DMI ratio on the % yield (GC) to **3**. 2.5 mmol DMI, no catalyst, no solvent, 21 °C, monitored by GC‐FID over 72 h. [Color figure can be viewed at http://wileyonlinelibrary.com]

To identify the effect of increased amine concentration on selectivity and isomerisation, several DEA:DMI molar ratios were investigated (Fig. 2). The largest change in conversion was seen when increasing the DEA:DMI ratio from 2:1 to 4:1, with conversions almost doubling over time. Further increase in DEA concentration had a reduced positive effect, particularly when comparing 6:1 to 8:1.

**Figure 3:**
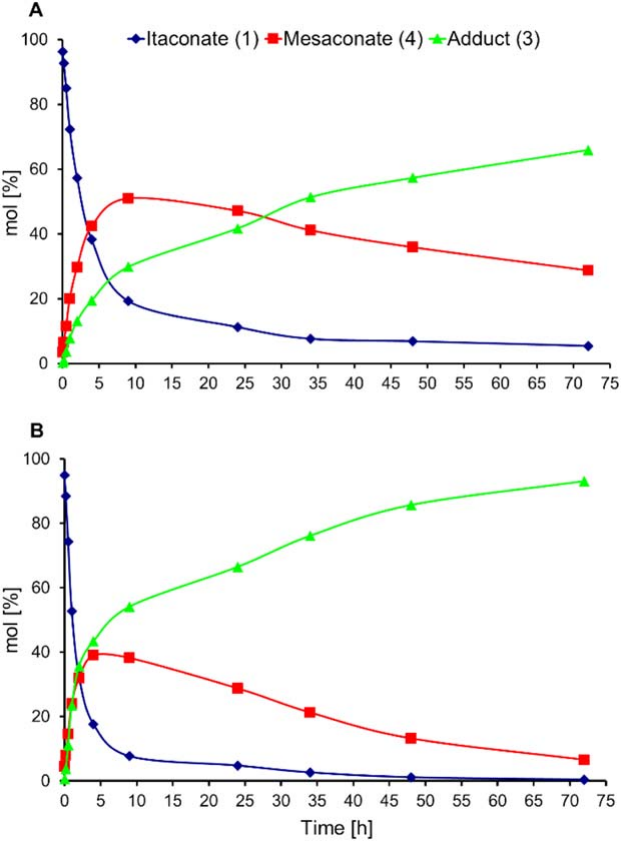
Reaction profiles for the DEA addition to DMI. DEA:DMI ratios of 2:1 (A, top) and 8:1 (B, bottom). 2.5 mmol DMI, 5 (A) or 20 mmol (B) of DEA. No catalyst, no solvent, 21 °C. [Color figure can be viewed at http://wileyonlinelibrary.com]

Upon closer inspection of the reactions profiles, it was clear that a number of equilibriums existed between the different compounds **1–5**, which appeared to change over time. As shown by the reaction profiles (Fig. 3), the consumption of the DMI **1** starting material proceeds by two competing reactions; the desired aza‐Michael addition (forming compound **3**) or isomerisation to mesaconate **4**. Negligible quantities of citraconate **5** were detected by GC or ^1^H NMR spectroscopy, showing that **4** is the dominant isomer. Initial consumption of DMI **1** was rapid, and increased in conjunction with the DEA:DMI ratio, shown in Figure 3(B). The maximum observed amount of **4** was seen for the lowest DEA:DMI ratio (2:1). This was anticipated, as isomerisation was in competition with aza‐Michael addition, with the addition logically becoming more favourable for the higher amine concentrations. Interestingly despite the ability of isomer **4** to undergo aza‐Michael addition, only product **3** rather than **6** or **7** was detected.

**Figure 4:**
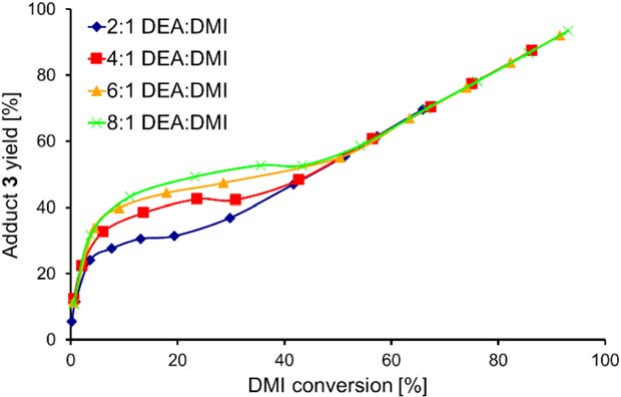
Effect of changing % selectivity to **3** versus % conversion of DMI. 2.5 mmol DMI, no catalyst, no solvent, 21 °C, varied DEA:DMI molar ratio. [Color figure can be viewed at http://wileyonlinelibrary.com]

Conversely, a higher selectivity to adduct **3** rather than isomer **4** was observed when using higher ratios of DEA. At higher DEA:DMI [Fig. 3(B)], DMI was consumed faster by the addition reaction, leaving less time for the competing isomerisation to **4**. It was also observed that, despite the conversion versus selectivity trends for altered DEA:DMI ratio initially being different, after ∼55% conversion the trends all appeared to align (Fig. 4). It was initially considered possible that the product acted as a basic catalyst in the reaction at conversions >55%. However, upon adding triethylamine to the system, it was found the tertiary amine has no catalytic ability for addition (see Supporting Information Fig. S3). It is possible that the excess DEA present was an effective enough catalyst itself that no benefit from TEA was observed. Interestingly when using 100%mol TEA the yield of adduct **3** dropped, this we assumed (and confirmed later) was a result of TEA also catalysing the isomerisation and creating more of the slower reacting **4**.

**Scheme 4:**
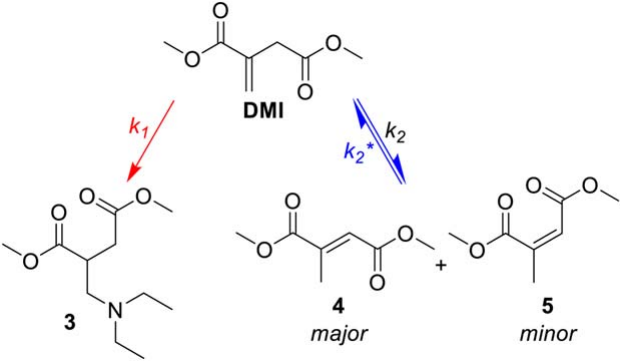
Competition between additions of DEA to DMI to form adduct **3** or isomerisation of DMI to mesaconate (**4**) and citraconate (**5**). [Color figure can be viewed at http://wileyonlinelibrary.com]

An alternative reasoning for the alignment after 55% conversion in Figure 4 was that the rate determining step at conversions below 55% was the aza‐Michael addition (*k*_1_, Scheme 4), while above a 55% conversion was rate‐determined by the regioisomerisation of **4** (formed in the earlier stages of the reaction) back to DMI, prior to its aza‐Michael addition (*k*_2_*, Scheme 4).

**Figure 5:**
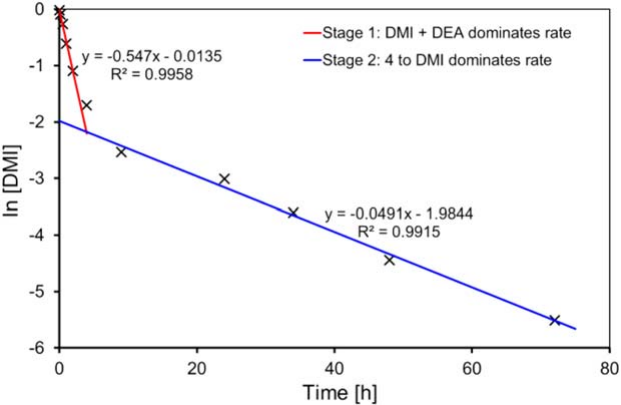
Plot of ln[DMI] versus time [h] for an 8:1 DEA:DMI ratio, highlighting the two separate rates evident in the Michael addition of DEA to DMI. 2.5 mmol DMI, 20 mmol DEA, no catalyst, no solvent, 21 °C. [Color figure can be viewed at http://wileyonlinelibrary.com]

The data gathered for the reaction profiles above was used to tentatively assess the kinetics of the system. The reaction was not performed in a solvent, therefore changing the DEA:DMI ratio also changed the *t*_0_ concentration of DMI and as such, only a rough assessment of the kinetics is possible. Further to this, DEA was not in adequate excess to be certain that the pseudo first‐order principle was applicable to the DMI starting material. Nevertheless, when plotting the relevant graphs to determine the order of reaction with respect to DMI in the 8:1 system (Supporting Information Fig. S4), it was evident that the reaction has two distinct stages (Fig. 5) that are both 1st order to [DMI]. It is postulated that, stage 1 (0–2 h) is where the aza‐Michael addition of DEA to itaconate is the rate determining step *k*_1_ (red line), and that stage 2 (9–72 h) is where rate of formation of **3** is determined by *k*_2_ * isomerisation of **4** to DMI (blue line). As such, the initial gradient is a combination of rates for consumption of DMI through both isomisation and the aza‐Michael addition rate of reaction, where the later gradient instead reflects the formation of DMI from **4** (*k*_2_ *). This would also agree with the reaction profile for the 8:1 DEA:DMI experiment, which seems to clearly indicate rapid formation of **3** within the first 2 h, slowing dramatically for the remainder of the experiment [Fig. 3(B)].

By splitting the kinetic data into two‐stages (0–2 and 9–72 h), 1st order with respect to [DMI] was confirmed to be appropriate for the majority of the other DEA:DMI ratios (Table 1, see Supporting Information Figs. S5 and S6 for all plots). However, for the 2:1 DEA:DMI system 1st order with respect to [DMI] displayed non‐linear behaviour for stage 2 of the reaction (Supporting Information Fig. S6, top left). When considering the proposed progression of the reaction (Scheme 4), this observation would be anticipated, as the second stage of the reaction should instead be controlled by [**4**] and not [DMI]. This becomes especially relevant if we assume that the addition of DEA to DMI is faster than isomerisation of **4** to DMI, as is indicated by both the reaction profiles in Figure 3 and the kinetic data in Table 1. We therefore assessed the reaction order for stage 2 with respect to [**4**], in all cases finding this also gives a near linear correlation for 1st order, and is certainly more appropriate for the lower DEA:DMI molar ratios. The other plots for assessment of the order and *k*_obs_ are available in Supporting Information Figure S7.

**Table 1:** *k*_obs_ for the DEA + DMI Aza‐Michael Addition, with Various DEA:DMI Molar Ratios and 1st‐Order Kinetics w.r.t. [DMI]

DEA:DMI Ratio	Stage of Reaction	*k*_obs_ × 10^−6^/s^−1^	*R* ^2^ of 1st‐Order Plot	aRate at *t*_0_ × 10^−6^/mol L^−1^ s^−1^
2:1	1 (0–2 h)	73.2	0.9964	202.8
	2 (9–72 h)	5.4	0.8677	2.8
	2^b^	2.6	0.9896	3.8
4:1	1	108.1	0.9976	288.1
	2	9.3	0.9923	3.0
	2^b^	5.8	0.9937	7.9
6:1	1	121.5	0.9976	297.0
	2	11.8	0.9944	2.6
	2^b^	7.4	0.9938	8.7
8:1	1	151.9	0.9958	316.1
	2	13.6	0.9915	2.7
	2^b^	8.0	0.9921	8.3

2.5 mmol DMI, varying amount of DEA, no catalyst, no solvent, 21 °C.

a Rate = −d[DMI]/d*t* = *k*_obs_[DMI]*t*_0_ for when 1st‐order w.r.t. [DMI]; rate = −d[**4**]/d*t* = *k*_obs_[**4**]t_0_ for when 1st‐order w.r.t. [**4**].

b 1st‐order kinetics w.r.t. [**4**] instead of [DMI]. The data point at 4 h was omitted from the calculations as a transitional period between the two stages of the reaction.

The rate of reaction for stage 1 was up to nearly one order of magnitude faster than for stage 2 for each ratio DEA:DMI, thus further supporting our hypothesis of stage 2 being slower and controlled by [**4**]. This demonstrates the significance that isomerisation of DMI to **4** as a competing reaction when attempting the aza‐Michael addition onto itaconates, and also the negative implication this isomerisation can have on yields of the addition for short reaction times. An important consideration is that DMI itself remains stable to isomerisation over long periods of storage in its liquid state, and that therefore formation of isomer **4** must require the presence of a catalyst. We therefore assumed that DEA additionally acts as a catalyst for this isomerisation. As such we investigated if basic but non‐nucleophilic amines could bring about the isomerisation. Indeed, we found that refluxing DMI in triethylamine for 24 h readily promoted the isomerisation to **4**, thus supporting the case that an amine base can indeed catalyse the regioisomerisation.

### Mesaconate:Itaconate 3:1 Starting Material

**Figure 6:**
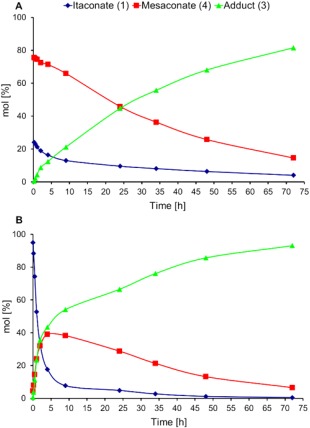
Effects of changing from a **4**:DMI 3:1 starting material (A) to a solely DMI (**1**) starting material (B) on the reaction profiles for the aza‐Michael addition of DEA to DMI. 2.5 mmol of 3:1 **4**:DMI (A) or DMI (B), 20 mmol DEA, no catalyst, no solvent, 21 °C. [Color figure can be viewed at http://wileyonlinelibrary.com]

To confirm further that the slower rate of stage 2 was due to isomerisation of **4** to DMI, a mixture of **4** and DMI (∼3:1 ratio, see Supporting Information Figure S9, confirming no citraconate observed) was synthesised (using the aforementioned triethylamine protocol to reach the thermodynamic equilibrium of the isomerisation). This was intended to mimic the reaction mixture at a later stage where conversion of **4** to DMI was the rate determining step for the formation of **3**. A comparison of the reaction profiles (Fig. 6) demonstrated an obvious difference in the initial rate of formation of **3** (green line), with the **4**:DMI equilibrium [Fig. 6(A)] clearly slowing the formation of the aza‐Michael product (**3**) from the outset.

**Figure 7:**
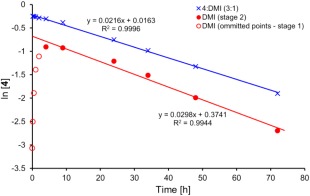
Effects of changing from DMI (red) to **4**:DMI 3:1 (blue) starting material on the plot of ln[**4**] versus reaction time [h]. 2.5 mmol DMI (red) or 3:1 **4**:DMI (blue), 20 mmol DEA, no catalyst, no solvent, 21 °C. [Color figure can be viewed at http://wileyonlinelibrary.com]

As is shown by the ln[**4**] versus time plots in Figure 7, trends for the DMI (blue line) and 3:1 **4**:DMI (red line) starting material systems have similar gradients for the later stages of reaction (>4 h). The parallel trend confirming the proposed theory that the slower rate for the second stage of the reaction was indeed due to the equilibrium between **4** and DMI controlling rate of formation of **3**.

All DEA:acceptor ratios agreed with a similar *k*_obs_ when replacing the DMI with the equilibrated 3:1 **4**:DMI (Table 2). The *k*_obs_ for the **4**:DMI system was found to increase from 2:1 to 4:1 DEA:acceptor ratio (Supporting Information Fig. S8), but then did not increase further with increasing DEA. This may indicate that from 4:1 DEA:acceptor and above the required quantity of DEA needed to catalyse the isomerisation of **4** back to DMI was present, and again reiterates the role that DEA plays in promoting the isomerisation. This observation is reinforced when comparing % yield over time for the different DEA:acceptor ratios, with 4:1 and above all being nearly identical (Supporting Information Fig. S10). DEA's role as a catalyst may also account for the aforementioned non‐linear behaviour of 1st order [DMI] kinetic plots at low DEA:DMI ratios.

**Table 2:** Comparison of *k*_obs_ for the DEA + DMI (9–72 h, Stage 2) or DEA + 3:1 4:DMI (0–48 h, Single Stage) Addition, with Various DEA:DMI Ratios

DEA:Acceptor Ratio	Acceptor	*k*_obs_ × 10^−6^/s^−1^	*R* ^2^ of 1st‐Order Plot	aRate at *t*_0_ × 10^−6^/mol L^−1^s^−1^
2:1	DMI	2.6	0.9896	3.8
	3:1 **4**:DMI	4.6	0.9981	10.2
4:1	DMI	5.8	0.9937	7.9
	3:1 **4**:DMI	6.0	0.9970	8.1
6:1	DMI	7.4	0.9938	8.7
	3:1 **4**:DMI	6.3	0.9957	6.3
8:1	DMI	8.0	0.9921	8.3
	3:1 **4**:DMI	6.2	0.9967	4.9

2.5 mmol DMI or 3:1 **4**:DMI (acceptor), no catalyst, no solvent, 21 °C, *k*_obs_ determined from plot of ln[**4**] versus term (Supporting Information Fig. S8).

a Rate = −d[**4**]/d*t* = *k*_obs_[**4**]*t*_0_. *t*_0_ = 9 h for DMI as acceptor, *t*_0_ = 0 h for 3:1 **4**:DMI as acceptor.

**Figure 8:**
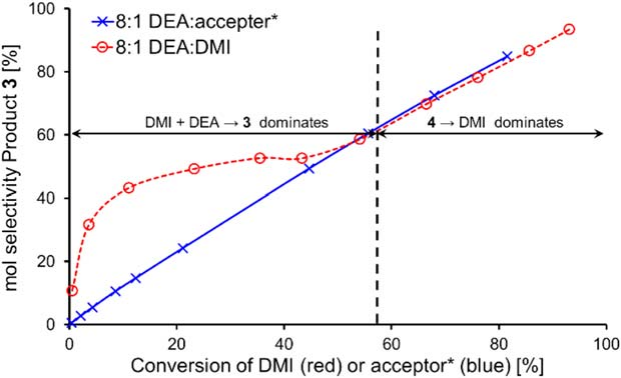
Comparison of % selectivity to compound **3** versus % conversion of DMI when starting from either DMI (red) or 3:1 **4**:DMMI (blue). 2.5 mmol DMI (red) or 3:1 **4**:DMI (blue), 20 mmol DEA, no catalyst, no solvent, 21 °C. [Color figure can be viewed at http://wileyonlinelibrary.com]

For the equilibrated system, plots of %conversion of diester versus %selectivity to **3** also showed no effect of DEA:DMI ratio, even at conversions below 55% (Supporting Information Fig. S11). However, it should be noted that the % selectivity for this plot is for **3** (adduct) versus **4** (isomer) and therefore **4** is being treated as both a product and a starting material. Nevertheless, superposition of the DMI starting material (exert from Fig. 4) on the **4**:DMI 3:1 plot, for conversion versus selectivity (Fig. 8) shows not only how the 0–55% region has been altered, but also that the trends have identical gradients above this key 55% conversion of DMI. This further supports the theory that the DMI to **4** equilibrium controlled the rate of formation of **3** in stage 2 of the reaction, and was the cause of the plot phenomenon originally observed in Figure 4, where the trends aligned at >55% conversion.

### Aza‐Michael Addition to Dimethyl Fumarate

**Scheme 5:**
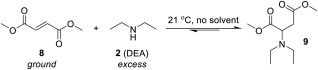
Aza‐Michael addition of DEA (**2**) to dimethyl fumarate (**8**) to form adduct **9**.

We remained intrigued as to why the regio‐selectivity for the above addition remained so high with regioisomer **4** seemingly never undergoing aza‐Michael addition to a detectable level despite using it as a starting material. We therefore extended the study to dimethyl fumarate (**8**, Scheme 5), to ascertain if its addition would prove to be as slow as for mesaconate **4**.

When initially applying the same methodology as above, we found **8** to be considerably less soluble in DEA compared to DMI or the 3:1 **4**:DMI mixture. As a result, all reactions with **8** typically contained solid material for the initial 24 h. Yields of adduct **9** improved when grinding **8** beforehand, due to the heterogeneous nature of the reaction. The product, **9**, was soluble in DEA and therefore as the reaction progresses the yields improved significantly, possibly as the presence of **9** aided dissolution of **8** in the solvent‐less system. This initial solubility issue means that assessment of kinetics was unreliable, and therefore only % yield (GC‐FID) of aza‐Michael adduct could **9** be used as a comparison (Table 3). Analogous to the DMI+DEA system, on increasing the amount of DEA the yield of adduct increased (Supporting Information Fig. S12), especially between 2:1 and 4:1, assumed to be a result of the aforementioned poor solubility of **8** being more pronounced at 2:1. From 24 h onwards for the 4:1 ratio and above, the fumarate system gave noticeably higher yields of the aza‐Michael adduct. Of significant importance is the comparison of the fumarate and the 3:1 **4**:DMI system, where yet again the drastic reduction in yield of adduct is clearly evident for the latter. This further supports the conclusion that the regioisomerisation associated with mesaconate **4** is responsible for detrimental slow rates of addition in the itaconate system.

**Table 3:** Comparison of %Yield of Aza‐Michael Adduct of DEA to Three Different Acceptor Systems (DMI, 3:1 4:DMI or 8) and Differing DEA:Acceptor Ratios

DEA:Acceptor Ratio	Acceptor	%Yield of Aza‐Michael Adduct^a^		
		2 h	24 h	48 h
2:1	DMI	13	42	57
	3:1 **4**:DMI	4	36	56
	**8** b	10	40	82
4:1	DMI	24	57	75
	3:1 **4**:DMI	7	42	66
	**8** b	17	90	97
6:1	DMI	29	63	82
	3:1 **4**:DMI	6	44	68
	**8** b	32	92	94
8:1	DMI	36	67	86
	3:1 **4**:DMI	9	44	68
	**8** b	42	92	93

2.5 mmol acceptor (DMI, 3:1 **4**:DMI or **8**), no catalyst, no solvent, 21 °C.

a %Yields (molar) were assessed a three different time intervals (2, 24, and 48 h) and determined by GC‐FID of the crude reaction mixture.

b **8** Ground with pestle and mortar prior to reaction.

Based on the itaconate system we had expected fumarate **8** to behave more like mesaconate **4**, and therefore result in lower yields. This was clearly not the case, but on reflection we attributed this to several possible reasons:

1. While itaconate **1** only possesses one site for attack from a soft Michael donor, fumarate **8** possesses two viable and equal positions of attack for soft nucleophiles, thus undergoing Michael addition at roughly double the rate (Fig. 9).
  **Figure 9:**
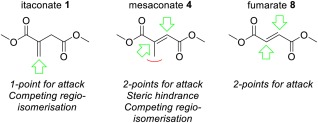
Possible positions for aza‐Michael addition attack on itaconate (**1**), mesaconate (**4**), and fumarate (**8**). [Color figure can be viewed at http://wileyonlinelibrary.com]
2. Mesaconate **4** also has two sites for addition, though one is sterically hindered by the methyl‐group (Fig. 9). This additional steric bulk may also promote the retro‐Michael addition for compounds **6** and **7** (Scheme 6), hence the reason they were not detected during our study.
  **Scheme 6:**
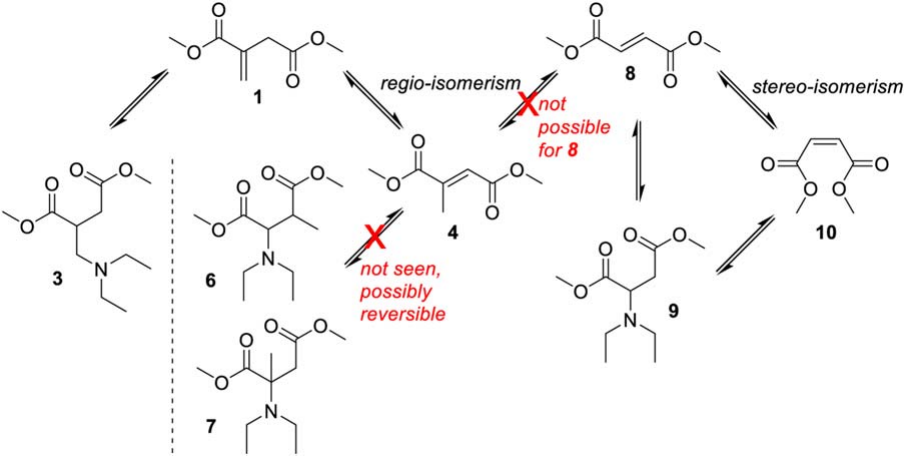
Comparison of itaconate (**1**) and fumarate (**8**) pathways to aza‐Michael adducts. [Color figure can be viewed at http://wileyonlinelibrary.com]
3. The C=C of **4** will also receive some electron density from the methyl group, this logically making it a less reactive with nucleophilic amine.
4. Fumarate **8** is unable to undergo C=C regioisomerisation (Scheme 6), a reaction that lowered the rate of formation of **3** by generating the less reactive isomer **4**. Although stereo‐isomerism is possible for **8** (to dimethyl maleate **10**) the aza‐Michael addition product from dimethyl maleate would also be **9** (Scheme 6).

Evidently, the behaviour of fumarate **8** in acting as an aza‐Michael acceptor clearly differentiates from itaconates, the key being the amine‐catalysed regioisomerisation of the latter dominating the rates of formation of adduct **3** during the later stages of the reaction. This study has therefore highlighted the fundamental issue in using itaconates, including polyesters, as an aza‐Michael acceptor and that if length of reactions need to be reduced then developing methods that promote the addition without also increasing regioisomerisation of the alkene are most certainly needed.

### Preliminary Studies of Addition to Itaconate Polyesters

**Scheme 7:**
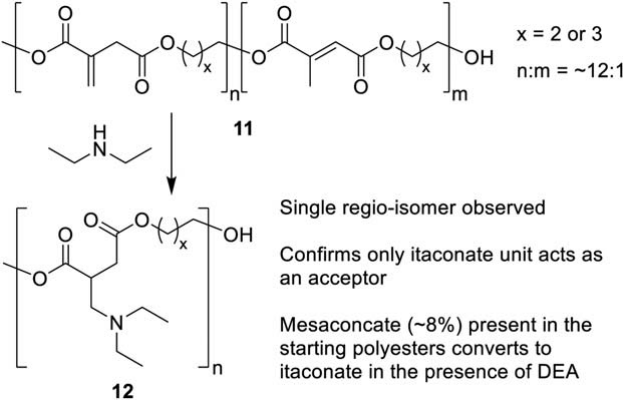
Comparison of itaconate (**1**) and fumarate (**8**) pathways to aza‐Michael adducts.

A significant observation from the above study was the remarkable regio‐selectivity of the addition, with only the itaconate reacting with DEA to form adduct **3**. As such we performed a preliminary study of DEA addition onto two itaconate polyesters (**11a** poly(1,3‐propylene itaconate) and **11b** poly(1,4‐butylene itaconate)). When leaving the solvent‐less reaction for an extended length of time (96 h) it was indeed confirmed that total regioselectivity with addition only to the itaconate (**12**, Scheme 7) was observed despite the starting unsaturated polyesters initially containing ∼8% mesaconate (see Supporting Information Figs. S13 and S14). This observation could highlight positive benefits for polymers modified in this manner as high regio‐selectivity leading to a single constitutional repeat unit will increase the likelihood of crystalline regions forming in modified polymer, possibly improving strength and stability of the resultant polyester.

## CONCLUSIONS

This comprehensive study of the aza‐Michael addition onto itaconates has shown how a competing pathway exists between the desired addition and the undesired regioisomerisation to the mesaconate unit. We have reaffirmed throughout this study that the mesaconate unit itself does not form the aza‐Michael adduct and that instead reformation of the itaconate from reversal of the regioisomerisation is required, this also leading to a single product (as confirmed by GC and NMR spectroscopy). The reversal of the regioisomerisation is roughly 1‐order of magnitude slower in rate than the initial addition to itaconate, and therefore extended reaction times of several days are required to allow near quantitative formation of the aza‐Michael adduct. By preparing an equilibrated itaconate:mesaconate mixture we confirmed both the role of amines as catalysts for the isomerisation and that the mesaconate does not form adduct but instead slowly reverts back to itaconate for subsequent addition to occur. The study was extended to fumarates and this highlight again the significant importance that the regioisomerisation plays, as formation of the fumarate adduct was significantly faster due to its inability to regio‐isomerise. When collectively reviewed the data presented here gives valuable insights into how aza‐Michael additions to itaconates should be carried out. The ideal target should be development of new catalysts that promote the desired Michael addition over the regioisomerisation, as the latter will inherently occur in the system due to the presence of amine. This conclusion is of particular relevance to researchers currently developing new polymers based on post‐polymerisation modification of itaconate polyesters, and would indicate that minimising regioisomerisation during formation of the polyester would also aid any subsequent post‐polymerisation step.

## EXPERIMENTAL

GC‐FID analysis was performed on an Agilent Technologies 6890N Network GC System (1 μL automated sample injection (Agilent Technologies 7683B Series Injector), 300 °C injector temperature, 40:1 split ratio, HP‐5 (5% phenyl methyl siloxane) capillary column (30 m × 0.32 mm, film thickness = 0.25 μm), 12 psi constant pressure, initial 2.5 mL min^−1^ He carrier gas flow rate). GC detection via an Aglient FID (300 °C FID heater temperature, 35 mL min^−1^ N_2_ make‐up gas, 35 mL min^−1^ H_2_, 350 mL min^−1^ air FID feed gas). ^1^H and proton‐decoupled ^13^C NMR spectra were obtained on a Jeol 400 spectrometer, in various solvents (CDCl_3_, D_2_O, *d*_6_‐DMSO or *d*_4_‐methanol). Chemical shifts were calibrated using the internal solvent resonance and referenced to TMS. IR spectra were obtained by running crude or purified samples neat on a Bruker Vertex 70 fitted with Specac Golden Gate ATR. Standard EI and CI GC–MS data were obtained using a HP 5890 capillary column gas chromatograph interfaced with a VG Autospec high performance mass spectrometer in positive ion mode. Accurate mass values were obtained using a Bruker MicrOTOF ESI‐TOF, and compared to theoretical 4.d.p relative molecular masses for assessment of product purity and elemental composition confirmation.

Room temperature aza‐Michael additions were performed in 5‐mL Supelco sealed glass multi‐position vessels, designed specifically for small scale reactions. The reactions were studied using GC‐FID analysis; DCM as the sample solvent and quench for reaction. To collect the crude product excess diethylamine (aza‐Michael donor) was removed under vacuum (2 mbar, >3 h). Purification of aza‐Michael addition product was achieved using a K60 packed column with a gradient solvent ratio (ethyl acetate/hexane). The purified products were used for determination of GC‐FID relative molar response factors (RMRFs) and assignment of retention times. Structures of products were confirmed by ^1^H and ^13^C NMR spectroscopy. RMRFs were determined (see Supporting Information Table S1 and Figure S2) for dimethyl itaconate (**1**) and dimethyl fumarate (**8**) and their corresponding aza‐Michael adducts (**3** and **9** respectively). RMRFs for dimethyl mesaconate (**4**) and dimethyl citraconate (**5**) were assumed identical to their regio‐isomer **1**, while the aza‐Michael adducts **6** and **7** were assumed to have the same RMRFs as their isomer **3**. RMRF for dimethyl maleate **10** was assumed identical to its stereo‐isomer **8**.

The suitability of chloroform as a solvent quench for the addition was also confirmed through NMR spectroscopy analysis. At each point of the kinetic study shown in Supporting Information Figure S15 a small amount (∼5 mg) of the reaction mixture were added in ∼1 mL of CDCl_3_ and the so prepared samples were run immediately on a Jeol JNM‐ECS400A to evaluate the kinetic of the reaction. The very same samples (stored in the NMR tube) were kept at 21 °C and analysed after 1, 2, 4, and 24 h from the initial ^1^H NMR spectroscopy analysis used for the kinetic study. Figure S16 in Supporting Information shows how, over the considered timeframe, there is no significant progression of the reaction, confirming that dilution of the reaction mixture in CDCl_3_ was an effective means of quenching for the purposes of the kinetic study.

Unsaturated polyesters for the preliminary study of the addition of DEA were prepared using the previously published method using a titanium alkoxide catalyst.^4^ ^(a)^ Addition to the polyesters was performed by dissolving 24 mmol (w.r.t. constitutional repeat unit) of polyester into 10.4 mL (100 mmol) of DEA and stirred at 21 °C for 96 h. The excess DEA was removed *in vacuo* under mild heating (65 °C) and the crude isolated polymer analysed by NMR spectroscopy (CDCl_3_ solvent). NMR spectra for isolated polyesters are shown in Supporting Information Figures S13 and S14.

### Preparation and Characterisation of 2‐(Diethylaminomethyl)‐Dimethyl Succinate) (3)

Dimethyl itaconate (4.7 g, 30 mmol) was dissolved in diethylamine (15.5 mL, 150 mmol) and stirred at room temperature for 5 days with completion of reaction confirmed by GC analysis. Excess diethylamine was removed *in vacuo* affording the desired product, compound **3** (2‐(diethylaminomethyl)‐dimethyl succinate), as a light brown slightly viscous liquid (6.7 g, 94%); diethylamine loss was observed on heating to temperatures greater than 80 °C; ^1^H NMR (400 MHz, CDCl_3_, *δ*_H_, ppm) 0.90 (6H, t, ^3^ *J* = 8.9 Hz, N(CH_2_C*H*_3_)_2_), 2.41 (6H, m, N(C*H*_2_CH_3_)_2_ and CHC*H*_2_CO_2_CH_3_)), 2.57 (2H, d, ^3^ *J* = 7.0 Hz, CHC*H*_2_N(CH_2_CH_3_)_2_), 2.96 (1H, dq, ^3^ *J* = 8.5 and 7.0 Hz, C*H*CH_2_CO_2_CH_3_), 3.60 (3H, s, CO_2_C*H*_3_), 3.62 (3H, s, CO_2_C*H*_3_); ^13^C NMR (100 MHz, CDCl_3_, *δ*_C_, ppm) 11.9 (2C, N(CH_2_ *C*H_3_)_2_), 34.3 (CH*C*H_2_CO_2_CH_3_), 40.9 (*C*HCH_2_CO_2_CH_3_), 47.1 (2C, N(*C*H_2_CH_3_)_2_), 51.7 (CO_2_ *C*H_3_), 51.8 (CO_2_ *C*H_3_), 54.7 (CH*C*H_2_N(CH_2_CH_3_)_2_), 172.8 (*C*O_2_CH_3_), 174.8 (*C*O_2_CH_3_); EI‐MS, 231 (molecular ion), 216, 200, 170, 159, 142, 127, 112, 99, 86 (100); EI‐MS accurate mass, 231.1466 (231.1471 calc. for C_11_H_21_NO_4_); IR (*ν*, cm^−1^), 2970 (C—H), 2808 (C—H), 1733 (C=O, ester), 1436 (H—C—H).

### Preparation of the Mixture Dimethyl Mesaconate:Dimethyl Itaconate:Dimethyl Citraconate (3:1 4:DMI)

Dimethyl itaconate (3.16 g, 0.02 moles) was dissolved in triethylamine (11.1 mL, 0.08 moles) and refluxed at 89 °C for 24 h, with the extent of isomerisation determined by GC‐FID and NMR analysis. Triethylamine was removed *in vacuo* affording a light brown product mixture with a DMMes:DMI:DMCit of 3:1:negligable (3.01 g, 74% DMMes); ^1^H NMR (400 MHz, CDCl_3_, *δ*_H_, ppm, DMI subtracted from spectra): 2.10 (3H, d, ^4^ *J* = 1.48 Hz, CH_3_CO_3_(C*H*_3_)C=CHCO_2_CH_3_), 3.58 (3H, s, CO_2_C*H*_3_), 3.62 (3H, s, CO_2_C*H*_3_), 6.58 (1H, q, ^4^ *J* = 1.48 Hz CH_3_CO_3_(CH_3_)=C*H*CO_2_CH_3_); ^13^C NMR (100 MHz, CDCl_3_, *δ*_C_, ppm): 13.8 (*C*H_3_ C=CHCO_2_CH_3_), 51.2 (CO_2_ *C*H_3_), 52.1 (CO_2_ *C*H_3_), 126.0 (CH_3_C=*C*HCO_2_CH_3_), 143.3 (CH_3_ *C*=CHCO_2_CH_3_), 165.8 (*C*O_2_CH_3_), 167.0 (*C*O_2_CH_3_). Analytical data was in agreement with the literature.^16^

### Preparation and Characterisation of 2‐(Diethylamino)‐Dimethyl Succinate (9)

Dimethyl fumarate (9.10 g, 63 mmol, ground with pestle and mortar) was dissolved in diethylamine (32.8 mL, 315 mmol) and stirred at room temperature for 3 days, with completion of reaction confirmed by GC analysis. Excess diethylamine was removed *in vacuo* affording the desired product, **9** (2‐(diethylamino)‐dimethyl succinate), as an orange to yellow slightly viscous liquid (11.4 g, 83%); diethylamine elimination (retro‐aza‐Michael addition) observed on heating to temperatures >80 °C; ^1^H NMR (400 MHz, CDCl_3_, *δ*_H_, ppm): 1.04 (6H, t, ^3^ *J* = 7.3 Hz, N(CH_2_C*H*_3_)_2_), 2.55 (5H, m, N(C*H*_2_CH_3_)_2_ and CHC*H*_2_CO_2_CH_3_)), 2.82 (1H, dd, ^3^ *J* = 7.0 and 15.7 Hz, CHC*H*_2_CO_2_CH_3_), 3.68 (3H, s, CO_2_C*H*_3_), 3.71 (3H, s, CO_2_C*H*_3_), 3.96 (1H, t, ^3^ *J* = 7.0 Hz, C*H*CH_2_CO_2_CH_3_); ^13^C NMR (100 MHz, CDCl_3_, *δ*_C_, ppm): 14.0 (2C, N(CH_2_ *C*H_3_)_2_), 34.7 (CH*C*H_2_CO_2_CH_3_), 45.0 (2C, N(*C*H_2_CH_3_)_2_), 51.5 (CO_2_ *C*H_3_), 51.7 (CO_2_ *C*H_3_), 59.1 (*C*HN(CH_2_CH_3_)_2_), 172.0 (*C*O_2_CH_3_), 172.7 (*C*O_2_CH_3_); ESI‐MS accurate mass, 218.1378 (MH^+^, 218.1387 calc. for C_10_H_20_NO_4_); IR (*ν*, cm^−1^), 2971 (C—H), 2844 (C—H), 1731 (C=O, ester), 1436 (H—C—H).

## ACCESS STATEMENT

All data used in the preparation of this manuscript for the sections funded by the EPSRC grant EP/L017393/1 is contained within this document, the electronic supplementary information, or available on request from https://doi.org/10.15124/3403a7f3-7967-4551-b4b7-f505c49ff2de.

## CONFLICT OF INTEREST

The authors declare no conflict of interest.

## Supporting information

Supporting information (SI) for this article is given via a link at the end of the document. SI contains additional analytical data; assessment of triethylamine as a catalyst; additional information for kinetic study; additional data for addition to fumarate; assessment of feasibility for solvent quench.
