# Supplementary material for: Insights into post‐polymerisation modification of bio‐based unsaturated itaconate and fumarate polyesters via aza‐michael addition: Understanding the effects of C=C isomerisation
Source: J Polym Sci A Polym Chem. 2018 Aug 9;56(17):1935–45. doi: 10.1002/pola.29079 (PMC6175416; doi:10.1002/pola.29079)
Supplement: Supplementary file 1 — Supporting information (SI) for this article is given via a link at the end of the document. SI contains additional analytical data; assessment of triethylamine as a catalyst; additional information for kinetic study; additional data for addition to fumarate; assessment of feasibility for solvent quench. [file POLA-56-1935-s001.pdf]

SUPPORTING INFORMATION

Insights into Post-polymerisation Modification of Bio-based Unsaturated Itaconate and Fumarate Polyesters via Aza-Michael Addition: Understanding the Effects of C=C Isomerisation

T. J. Farmer,* D. J. Macquarrie, J. W. Comerford, A. Pellis and J. H. Clark

Green Chemistry Centre of Excellence, Department of Chemistry, University of York, Heslington, York, United Kingdom, YO10 5DD

*thomas.farmer@york.ac.uk

Content of Supplementary Information

NMR Spectroscopy Analysis for Crude Compound **3** 2

GC-FID Relative Response Factors 3

Calculation of % Yield, Conversion and Selectivity from GC-FID Analysis 4

Assessment of Triethylamine as a Catalyst for Addition of DEA to DMI 5

Kinetic Plots for DMI + DEA reactions 5

NMR Spectroscopy Analysis of 3:1 Mixture of Mesaconate:Itaconate 8

Additional Assessment of DEA + DMMes:DMI (3:1) Reaction 9

Additional Analysis of Dimethyl Fumarate (**8**) and DEA Addition 10

Preliminary Study: Addition of DEA Onto Poly(1,3-propylene itaconate) 11

Preliminary Study: Addition of DEA Onto Poly(1,4-butylene itaconate) 12

Proof of Reaction Quench via Dilution with Chloroform 13

NMR Spectroscopy Analysis for Crude Compound 3

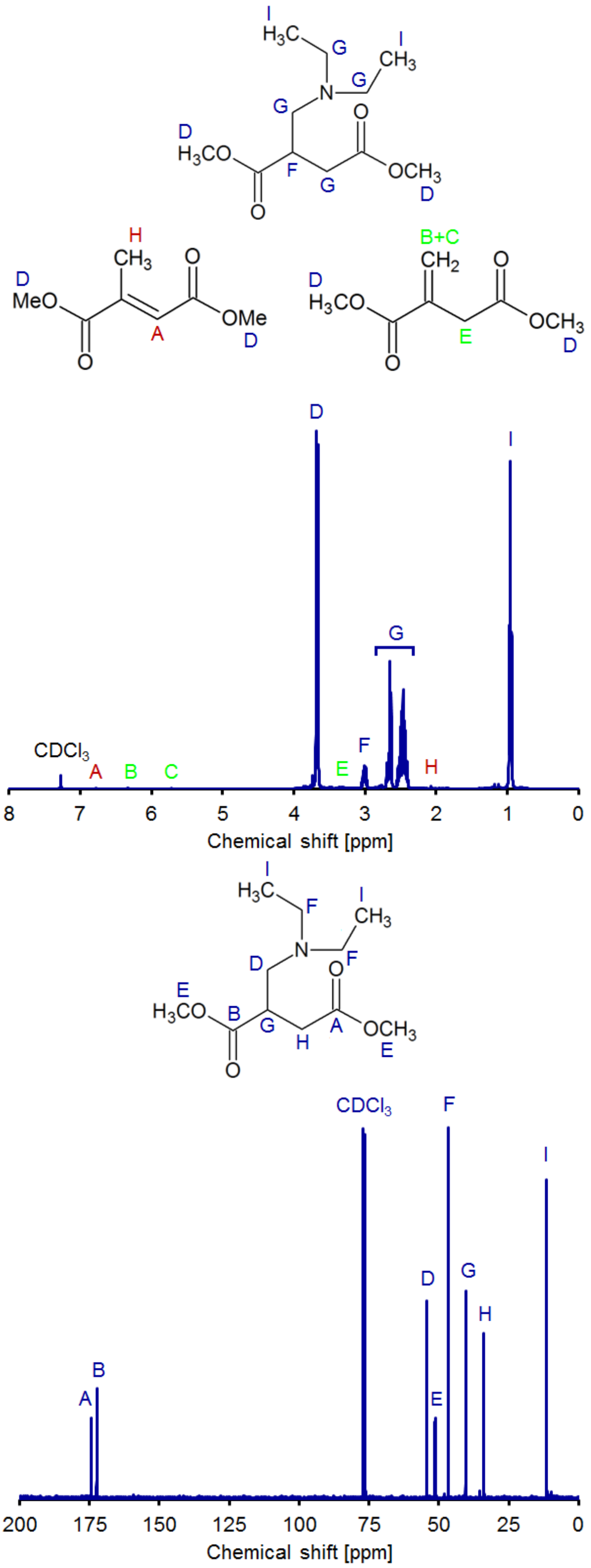

Figure S1 ^{1}H (top) and ^{13}C (bottom) NMR spectra for crude compound **3** in $CDCl_3$. DMI (4.7 g, 30 mmol) in DEA (15.5 mL, 150 mmol), 21 °C, 5 days, excess DEA removed *in vacuo* producing crude light brown slightly viscous liquid (**3**, 6.7 g, 94%). Signals for residual DMI and mesaconate (**4**) in ^{1}H spectra as A, B, C E, H

GC-FID Relative Response Factors

For reliable quantitative analysis using GC-FID, determination of relative molar response factors (RMRF) was required for the reactions studied. RMRFs were determined by running standard solutions of the Michael acceptors (itaconate and fumarate diesters) and the adducts (compounds **3** and **9**), of known concentrations. To avoid errors due to issues in the reproducibility of injections RMRFs were calculated from standard solutions containing all reaction components at varied relative ratios (e.g. Table S1). Gradients from plots of concentration versus GC-FID peak areas were compared for each component of a reaction, with the starting material taken to have a value of 1 and all other components a value relative to this (Figure S2). These values of relative response factors were incorporated into calculations of conversions, selectivity and yield. An example calculation is shown for compound **3** relative to DMI (**1**):

Solution Number	Solution Volume / ml	Molarityof DMI / mols l^{-1}	Molarity of Comp **3** / mols l^{-1}	Area of DMI peak	Area of Comp **3** peak
a	50	0.4	0	19904	0
e	50	0	0.4	0	32298
b	4	0.3	0.1	14777	8181
c	4	0.2	0.2	10773	15857
d	4	0.1	0.3	5647	22983

Table S1 Data used for determination of RMRF of compound **3** relative to DMI

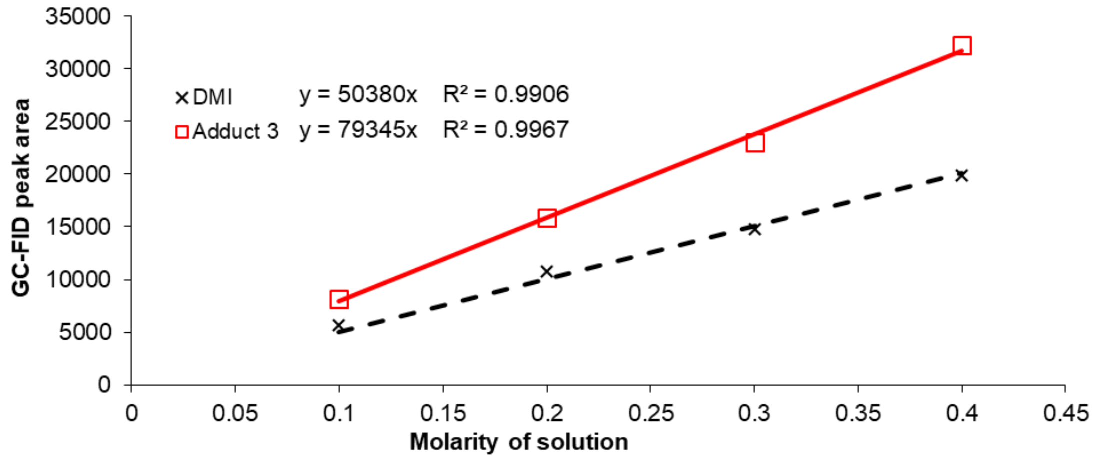

$RMRF_{Comp\ 3} = 79345 / 50380 = 1.57$

Figure S2 Determination of RMRF of compound **3** relative to DMI

The RMRF for dimethyl mesaconate (**4**) was assumed identical to its regio-isomer DMI. The RMRF of compound **9** relative to dimethyl fumarate (**8**) was determined to be 1.99.

Calculation of % Yield, Conversion and Selectivity from GC-FID Analysis

The calculations used throughout this investigation to assess the reactions are detailed in **Error! Reference source not found.** for a model reaction system. Relative molar response factor of **A** ($RMRF_A$) taken as 1. All other RMRFs calculated relative to $RMRF_A$**.** Peak area (PA) of GC response for each component measured from baseline.

For model reaction: **A** + **B** (excess) → **C (product)** → **D** + **E**

$$\%\ \text{Conversion of } \mathbf{A} = \frac{(PA_C / RMRF_C) + (PA_D / RMRF_D) + (PA_E / RMRF_E)}{PA_A + (PA_C / RMRF_C) + (PA_D / RMRF_D) + (PA_E / RMRF_E)} \times 100$$

$$\%\ \text{Selectivity to } \mathbf{C} = \frac{PA_C / RMRF_C}{(PA_C / RMRF_C) + (PA_D / RMRF_D) + (PA_E / RMRF_E)} \times 100$$

When mentioned the GC % yield refers to none-isolated value calculated from the GC trace, for example the GC % yield for **C** from the model system above is:

$$\%\ \text{Yield (GC) of } \mathbf{C} = 100 \times \frac{\%\ \text{selectivity to } \mathbf{C}}{100} \times \frac{\%\ \text{conversion of } \mathbf{A}}{100}$$

The isolated yield refers to the % of moles of purified product collected relative to the number of moles of starting material used, for the example above:

$$\%\ \text{Isolated yield of } \mathbf{C} = 100 \times \frac{\text{moles of purified product } \mathbf{C}}{\text{moles of starting material } \mathbf{A}}$$

For calculations of concentrations (see kinetic data), first the concentration of the starting material (e.g. DMI) was calculated at t0 using the known amounts of densities of each reagent, giving $[\mathbf{A}]_{t0}$. The concentration of the product (e.g. **C**) and the by-products (e.g. **D**) were calculated by multiplying their molar ratios (relative to A and all other products) by $[A]_{t0}$. These were then used to calculate kinetic data.

Assessment of Triethylamine as a Catalyst for Addition of DEA to DMI

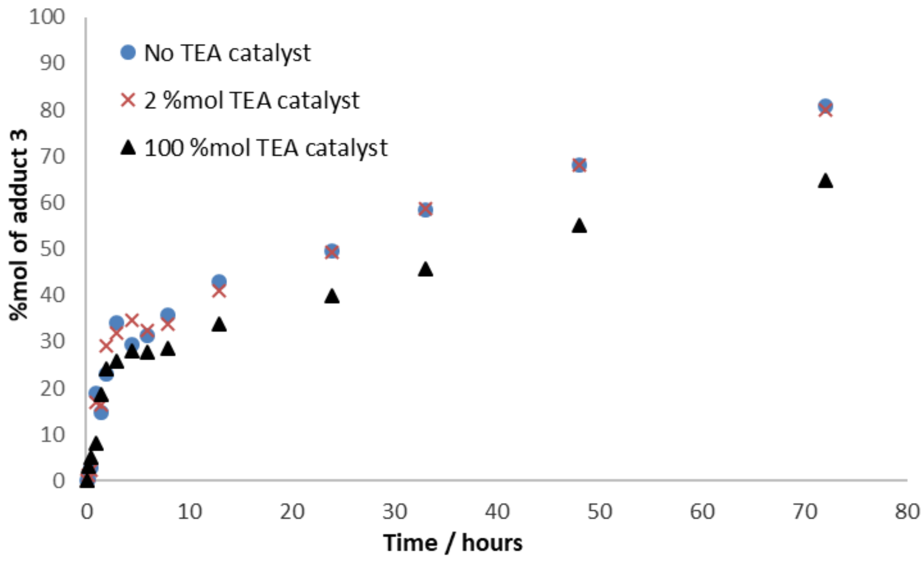

Figure S3 Assessment of triethylamine (TEA) as a catalyst (no TEA, 2%mol or 100%mol w.r.t. DMI) for the reaction of DEA and DMI. 2.5 mmol DMI, 20 mmol DEA, 0-2.5 mmol TEA, no solvent, 21 °C, GC-FID analysis.

Kinetic Plots for DMI + DEA reactions

Determination of order (for 8:1 DEA:DMI)

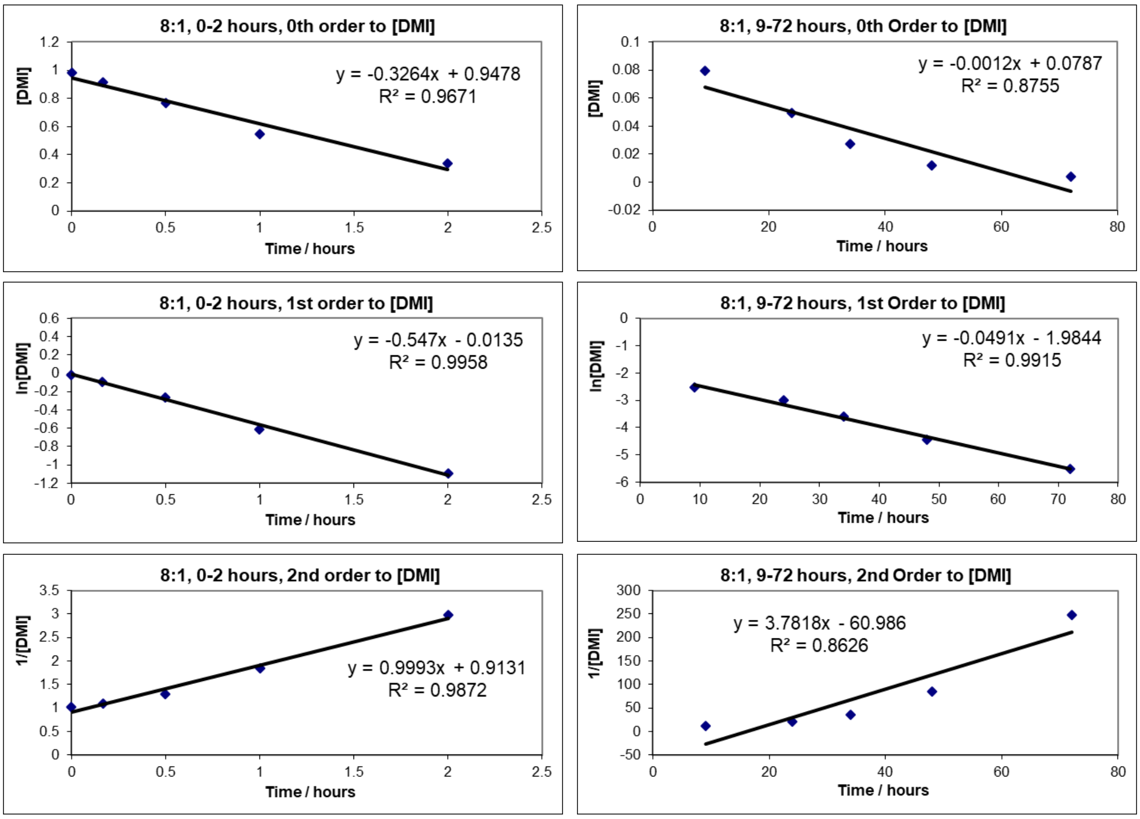

Figure S4 Various plots for the determination of the reaction order for 8:1 DEA:DMI system. Top to bottom = 0th to 2nd order; left to right = 0-2 hours and 9-72 hours. 2.5 mmol DMI, 20 mmol DEA, no catalyst, no solvent, 21 °C, GC-FID analysis

Determination of Stage 1 (0-2 hours) k_{obs} and R^2 for varied DEA:DMI where DMI is considered the rate-limiting reagent

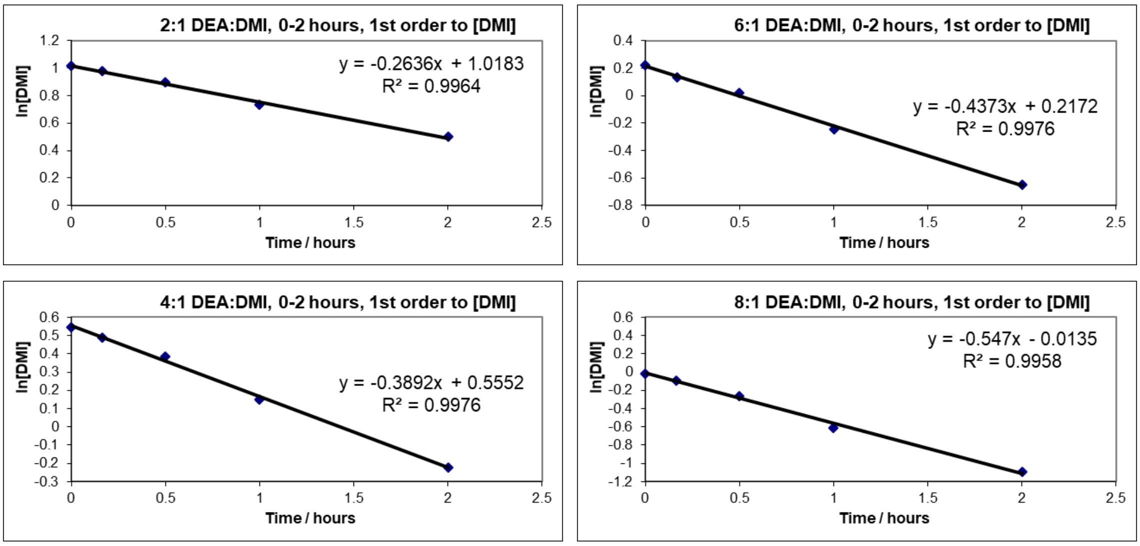

Figure S5 1st order plots (ln[DMI] vs time / hours) for varied DEA:DMI (2:1 to 8:1) for stage 1 (0-2 hours) of the reaction between DMI and DEA. 2.5 mmol DMI, 5-20 mmol DEA, no catalyst, no solvent, 21 °C, GC-FID analysis

Determination of Stage 2 (9-72 hours) k_{obs} and R^2 for varied DEA:DMI where DMI is considered the rate-limiting reagent

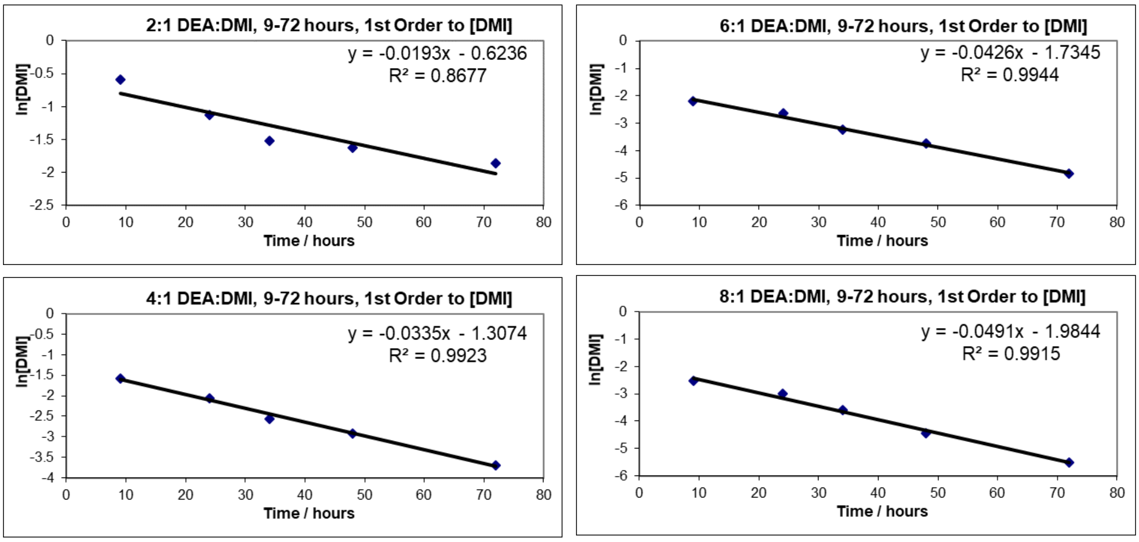

Figure S6 1st order plots (ln[DMI] vs time / hours) for varied DEA:DMI for stage 2 (9-72 hours) of the reaction between DMI and DEA

Determination of Stage 2 (9-72 hours) k_{obs} and R^2 for varied DEA:DMI where DMMes is considered the rate-limiting reagent

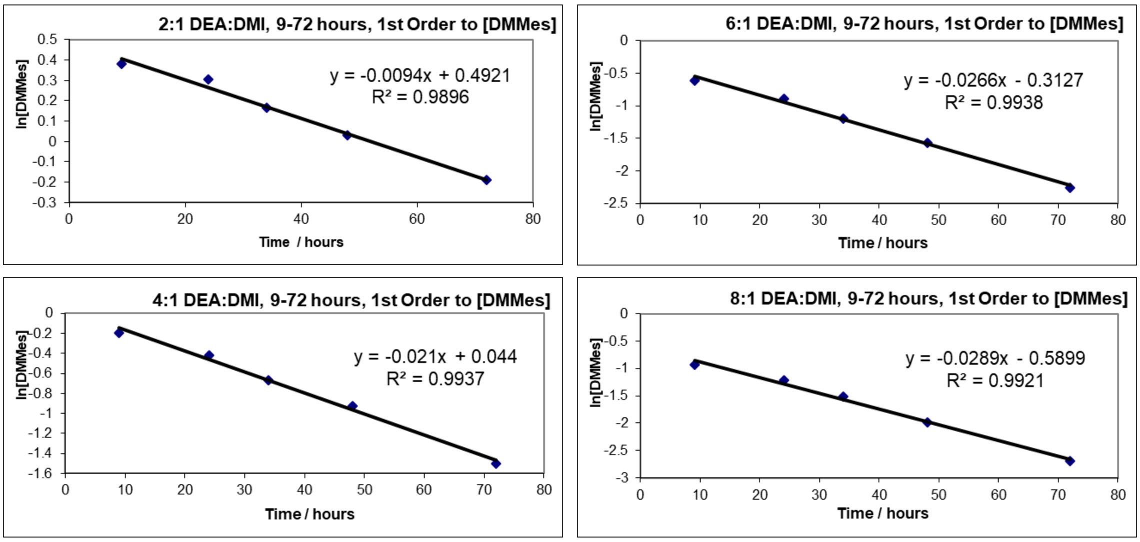

Figure S7 1st order plots (ln[DMMes] vs time / hours) for varied DEA:DMI for stage 2 (9-72 hours) of the reaction between DMI and DEA

Determination of k_{obs} and R^2 for varied DEA:acceptor where the acceptor is a 1:3 DMI:DMMes mixture and DMMes is considered the rate-limiting reagent

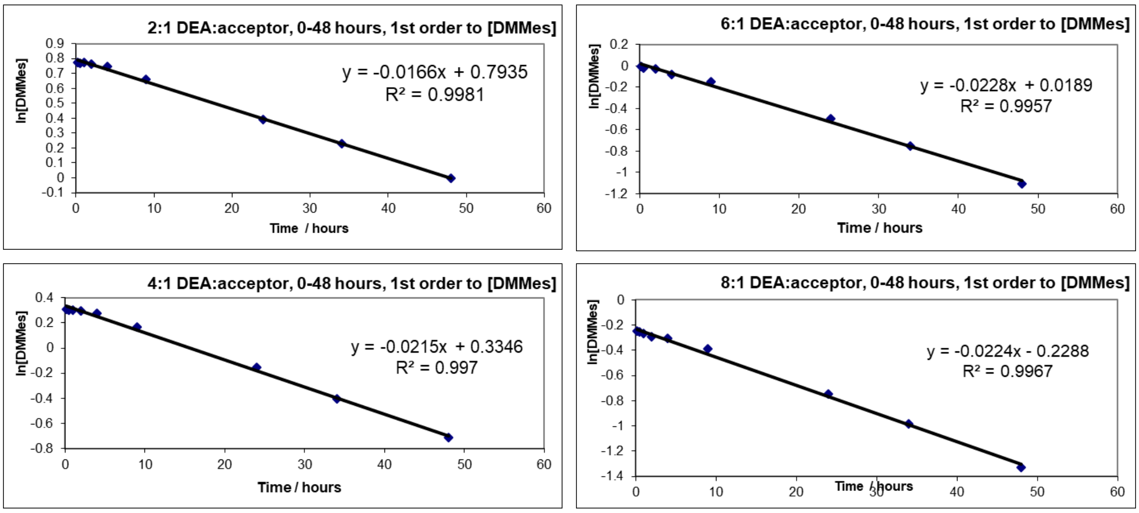

Figure S8 1st order plots (ln[DMMes] vs time / hours) for varied DEA:acceptor ratios (acceptor = 1:3 DMI:DMMes) for of the reaction between DMI:DMMes 3:1 and DEA. Note: 72 hour data point omitted due to non-linear behaviour for 8:1 system

NMR Spectroscopy Analysis of 3:1 Mixture of Mesaconate:Itaconate

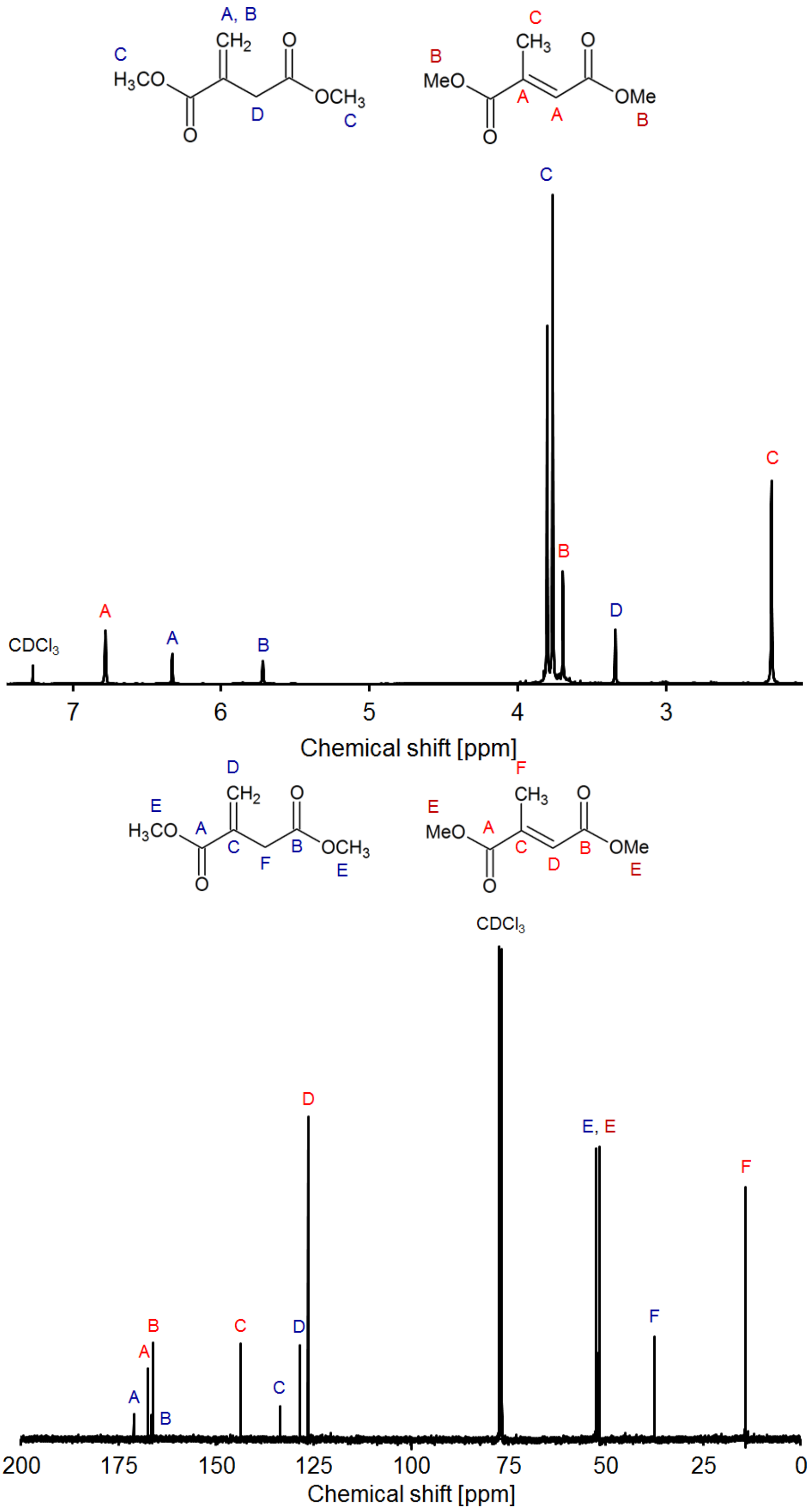

Figure S9 ^{1}H (top) and ^{13}C (bottom) NMR spectra for crude mixture of DMI:DMMes:DMCit (1:3:negligible) in $CDCl_3$. DMI (3.16 g, 0.02 moles) dissolved in TEA (11.1 ml, 0.08 moles) , 89 ˚C (reflux), 24 hours, TEA removed *in vacuo*.

Additional Assessment of DEA + DMMes:DMI (3:1) Reaction

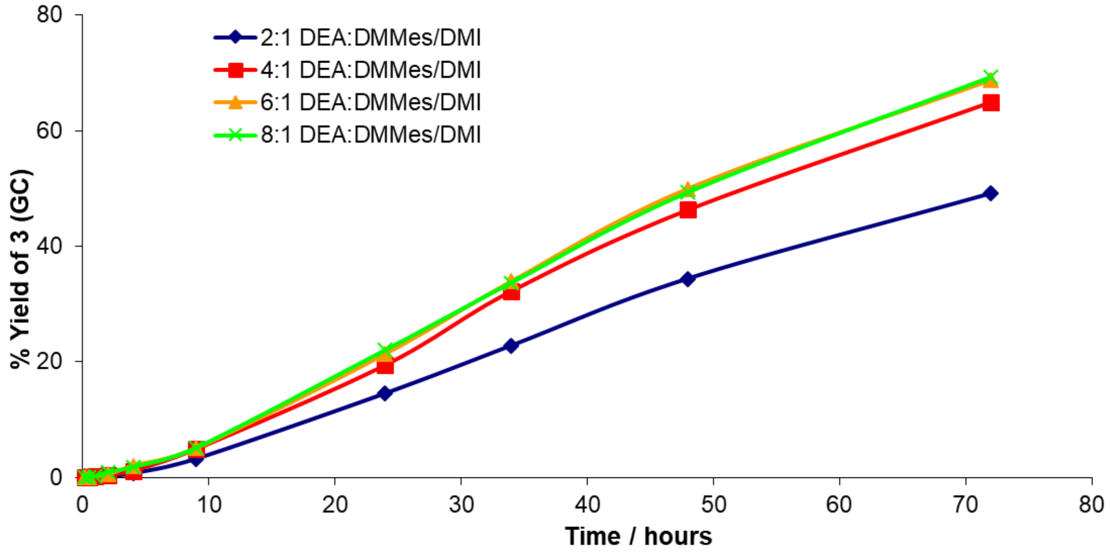

Figure S10 Effects of DEA:acceptor ratio of the % yield of compound **3** versus time when starting with 1:3 DMI:DMMes (acceptor). 2.5 mmol 1:3 DMI:DMMes, 5-20 mmol DEA, no catalyst, no solvent, 21 °C, GC-FID analysis

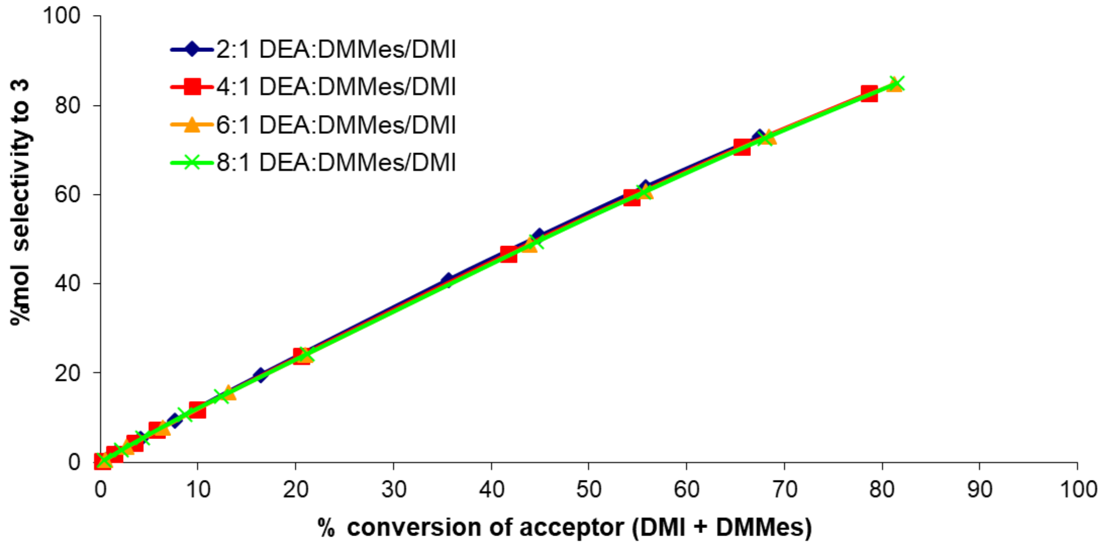

Figure S11 % Selectivity to compound **3** versus % Conversion of acceptor (DMI+DMMes) when starting with a 3:1 DMMes:DMI mixture. 2.5 mmol 3:1 DMMes:DMI, 5-20 mmol DEA, no catalyst, no solvent, 21 °C, GC-FID analysis

Additional Analysis of Dimethyl Fumarate (8) and DEA Addition

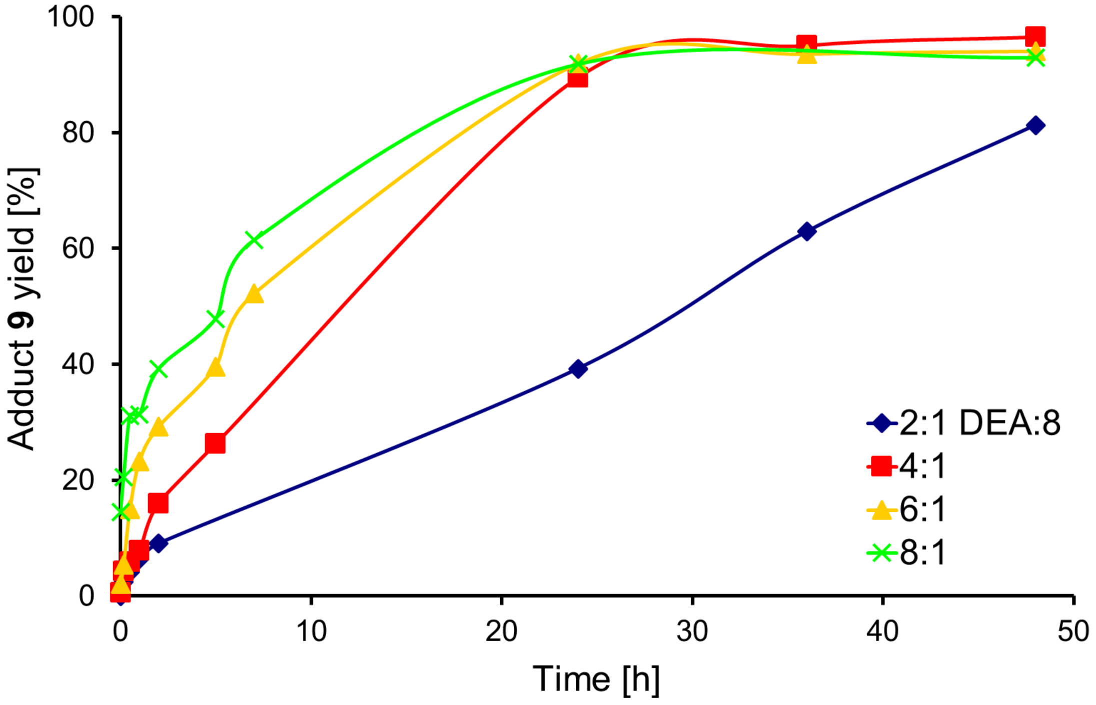

Figure S12 Effects of DEA:**8** ratio on the % yield (GC) of compound **9** over time for the aza-Michael addition of DEA to **8**. 2.5 mmol ground **8**, variable amount of DEA, no catalyst, no solvent, 21 ºC

Preliminary Study: Addition of DEA Onto Poly(1,3-propylene itaconate)

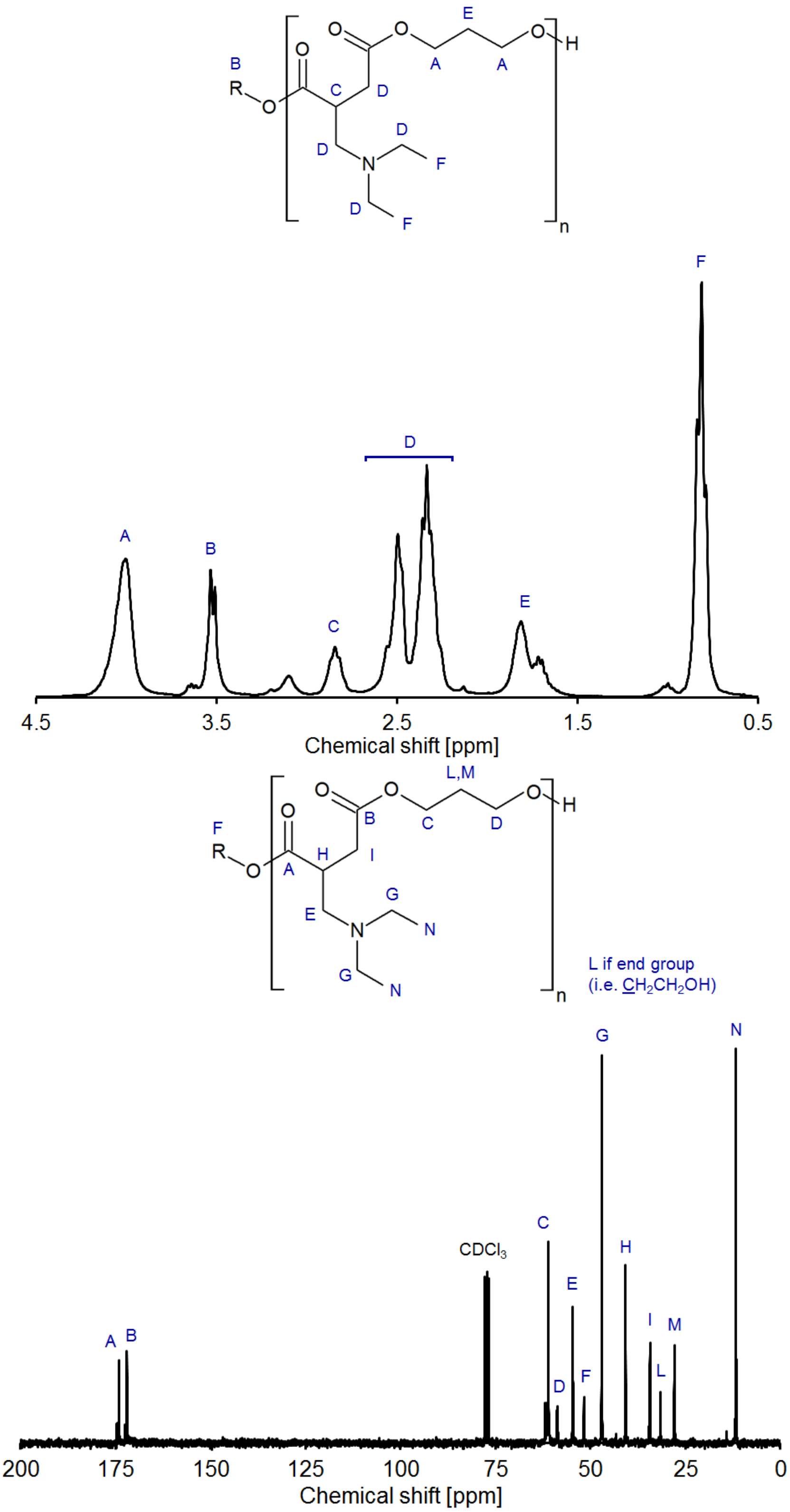

Figure S13 ^{1}H (top) and ^{13}C (bottom) NMR spectra for crude isolated polyester from the addition of DEA to poly(1,3-propylene itaconate) in $CDCl_3$. Poly(1,3-propylene itaconate) (4.08 g, 24 mmol), DEA (10.4 ml, 100 mmol), 21 ˚C, 96 hours, excess DEA removed *in vacuo* (65 °C). No evidence of addition to mesaconate unit.

Preliminary Study: Addition of DEA Onto Poly(1,4-butylene itaconate)

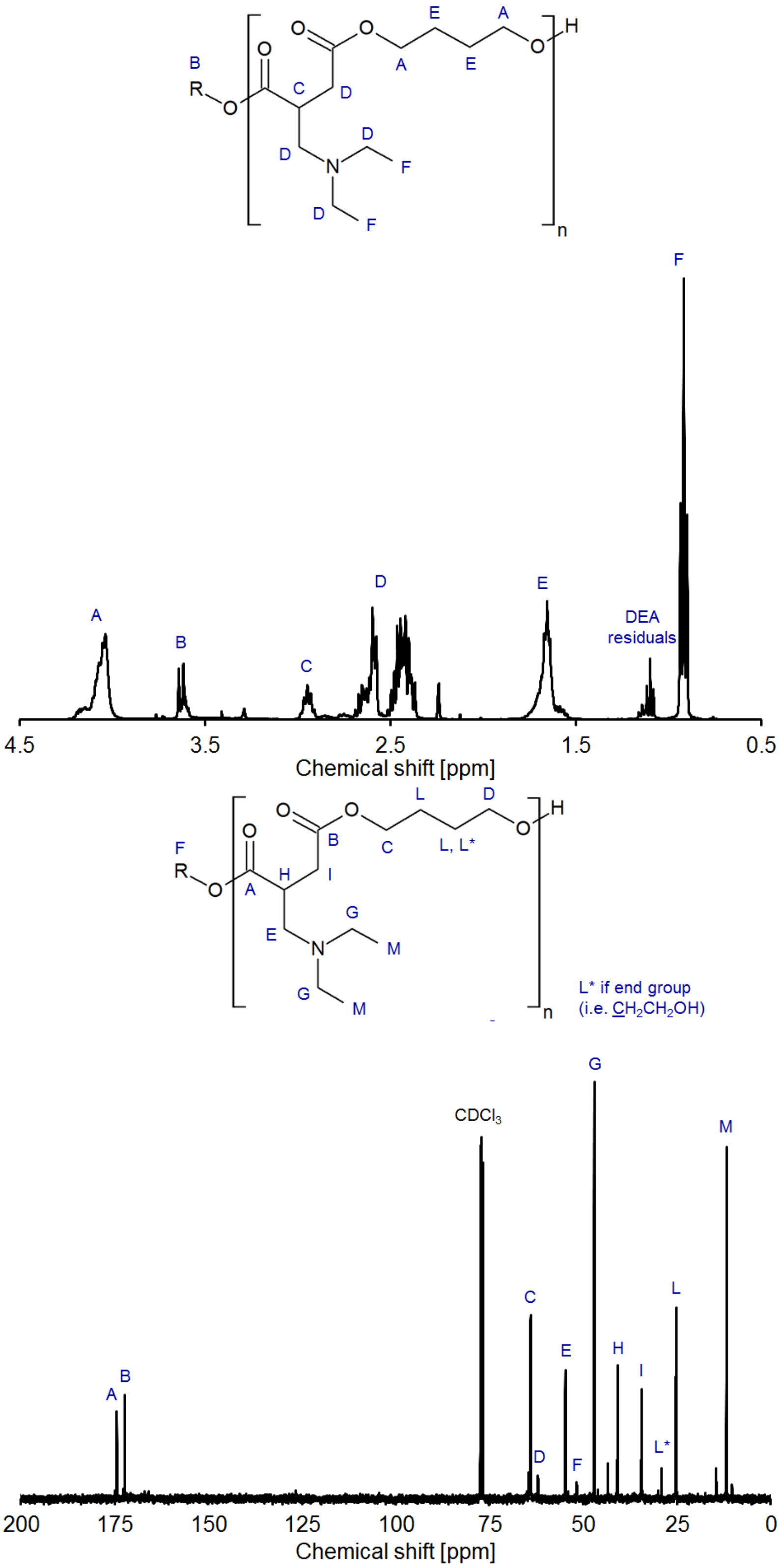

Figure S14 ^{1}H (top) and ^{13}C (bottom) NMR spectra for crude isolated polyester from the addition of DEA to poly(1,4-butylene itaconate) in $CDCl_3$. Poly(1,4-butylene itaconate) (4.42 g, 24 mmol), DEA (10.4 ml, 100 mmol), 21 ˚C, 96 hours, excess DEA removed *in vacuo* (65 °C). No evidence of addition to mesaconate

Proof of Reaction Quench via Dilution with Chloroform

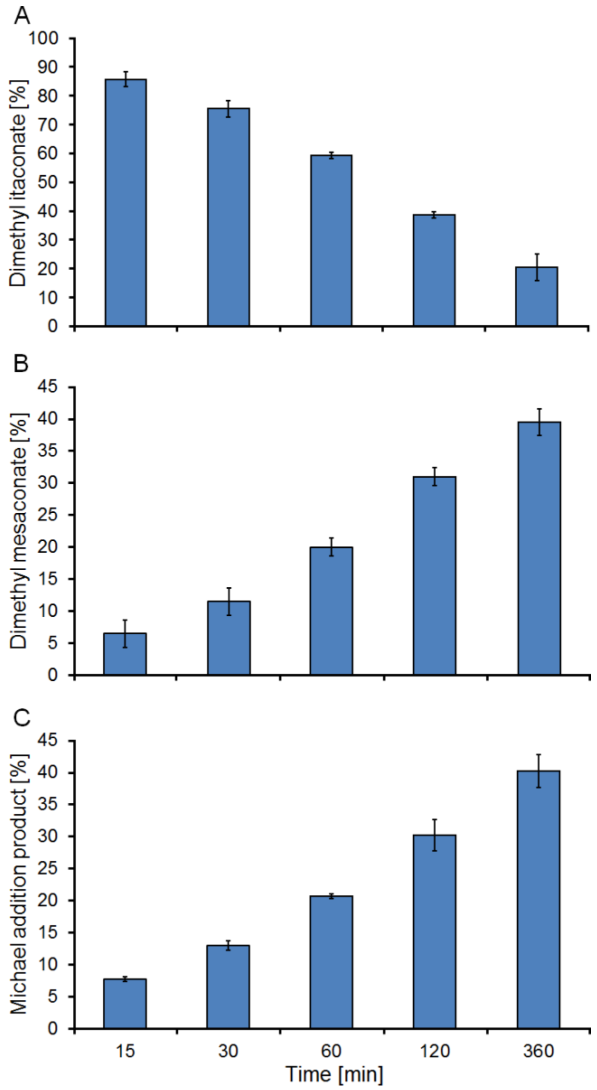

Figure S15 Time course reaction of the Michael addition of DEA (10 mmol) on DMI (2.5 mmol). DEA:DMI ratio= 4:1. The reaction was followed over time via ^{1}H-NMR. A) % DMI in the reaction mixture; B) %DMM in the reaction mixture and C) % of the Michael addition product in the reaction mixture. All experiments were performed in duplicates.

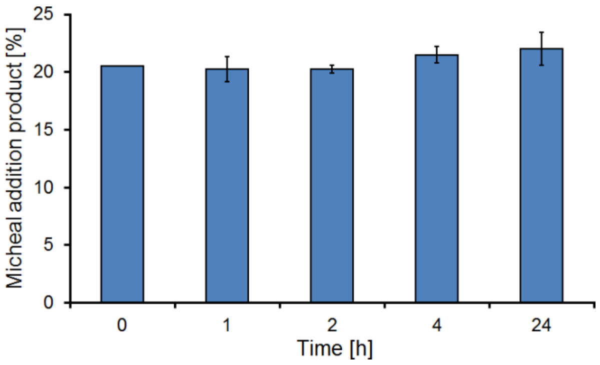

Figure S16 Re-run of NMR samples (from 60 min above) over a 24 h period. ~5 mg of the reaction mixture (60 min), ~1.0 mL of $CDCl_3$. Conversions monitored over time via ^{1}H-NMR spectroscopy. All experiments performed in duplicates.
